# HK2 Is a Crucial Downstream Regulator of miR-148a for the Maintenance of Sphere-Forming Property and Cisplatin Resistance in Cervical Cancer Cells

**DOI:** 10.3389/fonc.2021.794015

**Published:** 2021-11-11

**Authors:** Hao Yang, Hui Hou, Haiping Zhao, Tianwei Yu, Yuchong Hu, Yue Hu, Junmei Guo

**Affiliations:** ^1^ Department of Radiation Oncology, Inner Mongolia Cancer Hospital and Affiliated People’s Hospital of Inner Mongolia Medical University, Hohhot, China; ^2^ Department of Pediatric Hematology and Oncology, Inner Mongolia Autonomous Region People’s Hospital, Hohhot, China; ^3^ Department of Abdominal Tumor Surgery, Affiliated Hospital of Inner Mongolia Medical University, Hohhot, China; ^4^ Department of Transfusion Medicine, Inner Mongolia Cancer Hospital and Affiliated People’s Hospital of Inner Mongolia Medical University, Hohhot, China; ^5^ Department of Gynaecology, Inner Mongolia Autonomous Region People’s Hospital, Hohhot, China

**Keywords:** HK2, miR-148a, sphere formation, cisplatin resistance, cervical cancer

## Abstract

The acquisition of cancer stem-like properties is believed to be responsible for cancer metastasis and therapeutic resistance in cervical cancer (CC). CC tissues display a high expression level of hexokinase 2 (HK2), which is critical for the proliferation and migration of CC cells. However, little is known about the functional role of HK2 in the maintenance of cancer stem cell-like ability and cisplatin resistance of CC cells. Here, we showed that the expression of HK2 is significantly elevated in CC tissues, and high HK2 expression correlates with poor prognosis. HK2 overexpression (or knockdown) can promote (or inhibit) the sphere-forming ability and cisplatin resistance in CC cells. In addition, HK2-overexpressing CC cells show enhanced expression of cancer stem cell-associated genes (including *SOX2* and *OCT4*) and drug resistance-related gene *MDR1*. The expression of HK2 is mediated by miR-145, miR-148a, and miR-497 in CC cells. Overexpression of miR-148a is sufficient to reduce sphere formation and cisplatin resistance in CC cells. Our results elucidate a novel mechanism through which miR-148a regulates CC stem cell-like properties and chemoresistance by interfering with the oncogene *HK2*, providing the first evidence that dysregulation of the miR-148a/HK2 signaling plays a critical role in the maintenance of sphere formation and cisplatin resistance of CC cells. Our findings may guide future studies on therapeutic strategies that reverse cisplatin resistance by targeting this pathway.

## Introduction

Cervical cancer (CC) is ranked the fourth most commonly diagnosed cancer in women and is the second-leading cause of cancer-related death among women in developing countries (1). Surgery followed by a combination of platinum/paclitaxel-based chemotherapy is currently considered the standard of care for CC (2). Although the majority of CC patients have an initial response to chemotherapy, such as cisplatin (CDDP), a substantial proportion of patients eventually develop chemoresistance and relapse (2). Therefore, a better understanding of the mechanisms underlying CDDP resistance is required to retrieve the chemosensitivity in CC patients.

Most cancers contain a small subpopulation of cells (known as “cancer stem cells”) that drives the persistence of malignant tumors by producing new cancer cells (3). The cancer stem cell theory explains numerous clinical observations, such as the recurrence of tumors after initially successful chemotherapy and/or radiation therapy, and metastasis (3). Cancer stem cells have been found in many cancer types, including CC (4). In CC, cancer stem cells have been associated with resistance to commonly used anti-cancer drugs such as CDDP (4). Several potential stem cell markers, including MSI1, ALDH1, SOX2, and OCT4, have been used to identify putative cancer stem cells of CC (5). However, the molecular mechanisms underlying the induction and maintenance of cancer stem cells in CC remain to be explored.

In addition to several key genetic alterations in oncogenes and tumor suppressor genes, several epigenetic mechanisms, including microRNAs (miRNAs), have also been shown to regulate the progression and stemness of CC (6). Hexokinase 2 (HK2) is an enzyme that catalyzes the first committed step in glucose metabolism and converts glucose to glucose-6-phosphate (7). After knocking down HK2 expression, CC cells demonstrated significantly attenuated proliferation ability and glycolysis (8). Deletion of HK2 inhibits the growth and migration of CC cells (9). The same study has also demonstrated that knockdown of HK2 decreases the phosphorylation of AKT and mTOR, and increases the expression of p53 in CC cells (9). Another report indicated that HK2 promotes the proliferation of CC cells *in vitro* and tumor formation *in vivo* by regulating the Raf/MEK/ERK signaling pathway (10). Moreover, a previous study demonstrated that HK2 expression was suppressed by miR-9-5p by directly binding its 3’-untranslated region (3′-UTR) in CC cells (9). To the best of our knowledge, there have been no reports on the function and the underlying mechanism of HK2 in regulating CC stemness and CDDP resistance.

Here, we have explored for the first time that HK2 is overexpressed in CC, and its overexpression promotes sphere formation and CDDP resistance of CC cells. Moreover, we have demonstrated that the induction of HK2 expression in CC is partly due to the downregulation of miR-145, miR-148a, and miR-497. In particular, miR-148a acts as a key tumor suppressor that controls sphere formation and CDDP resistance by targeting HK2. Together, we have unveiled a novel epigenetic mechanism that controls sphere formation and CDDP resistance *via* the miR-148a/HK2 axis in human CC. Thus, therapeutic targeting this axis may reverse CDDP resistance in CC patients.

## Materials and Methods

### Human Tissue Specimens

This study was approved by the Research Ethics Committee of Affiliated Hospital of Inner Mongolia Medical University. Written informed consent was obtained from all patients. A total of 30 CC patients with no preoperative chemotherapy, immunotherapy, or radiotherapy were enrolled in this study. Samples were collected from patients who were diagnosed with CC and underwent surgical resection in the Affiliated Hospital of Inner Mongolia Medical University. After surgery, CC tissues and corresponding adjacent normal tissues were immediately frozen by liquid nitrogen and stored at -80°C for further analysis.

### Cell Lines and Cell Culture

Two human CC cell lines (HeLa and SiHa) were obtained from the American Type Culture Collection (ATCC, Manassas, VA). The HPV16 E6/E7-immortalized human cervical epithelial cell line Ect1/E6E7 (ATCC) was considered to be a non-cancerous ectocervical epithelial cell line (11, 12). All cells were cultured in DMEM/F12 medium (Sigma-Aldrich, St. Louis, MO) supplemented with 10% fetal bovine serum (FBS, Invitrogen, Carlsbad, CA) at 37°C with a humidified air containing 5% CO2. These cells were tested for mycoplasma contamination using the DAPI staining method.

### Quantitative Real-Time PCR (qRT-PCR) Analysis

Total RNA from CC cell lines and tissues was isolated using TRIzol (Invitrogen). Complementary DNA (cDNA) was obtained from 500 ng of the total RNA using the PrimeScript II cDNA Synthesis Kit (TaKaRa, Beijing, China). The qRT-PCR analysis was conducted with a PRISM 7700 (Applied Biosystems) using the Power SYBR Green PCR Master Mix (Invitrogen). The primers were as follows: *HK2*-F: 5′- GAGCCACCACTCACCCTACT-3′ and *HK2*-R: 5′- CCAGGCATTCGGCAATGTG-3′; *MDR1*-F: 5′- TTGCTGCTTACATTCAGGTTTCA-3′ and *MDR1*-R: 5′- AGCCTATCTCCTGTCGCATTA -3′; *SOX2*-F: 5′- GCCGAGTGGAAACTTTTGTCG-3′ and *SOX2*-R: 5′- GGCAGCGTGTACTTATCCTTCT-3′; *OCT4*-F: 5′-CTGGGTTGATCCTCGGACCT-3′ and *OCT4*-R: 5′-CCATCGGAGTTGCTCTCCA-3′; *GAPDH*-F: 5′-AATCCCATCACCATCTTC-3′ and *GAPDH*-R: 5′-AGGCTGTTGTCATACTTC-3′; U6-F: 5′-GCTTCGGCAGCACATATACTAAAAT-3′ and U6-R: 5′-CGCTTCACGAATTTGCGTGTCAT-3′. The NCode SYBR GreenER miRNA qRT-PCR analysis kit (Invitrogen) was used to validate the expression of miR-145, miR-148a, and miR-497 as previously reported (13). The expression of mRNA or miRNA was normalized to the expression of *GAPDH* mRNA or U6.

### Western Blotting Analysis

Total proteins were isolated from CC cells, which were lysed on ice with a RIPA lysis buffer (Cell Signaling Technology, MA). The protein concentration was determined using a BCA Protein Assay Kit (Beyotime Biotechnology, Shanghai, China). Extracted protein (30 µg) was separated by 12% SDS-PAGE and transferred onto a PVDF membrane (GE Healthcare Life Sciences, Piscataway, NJ). The membranes were blocked with 5% non-fat milk for 1 h and then incubated with anti-HK2 antibody (1:1000, #2106, Cell Signaling) and anti-GAPDH antibody (1:5000, #2118, Cell Signaling) at 4°C overnight. Then, the membranes were subsequently incubated with secondary antibodies for 2 h at room temperature. The protein bands were detected using an ECL detection kit (Amersham Pharmacia Biotech, UK).

### Sphere Formation Assay

Sphere formation assay was performed as previously described (6). CC cells (3000) cells were seeded in 6-well ultra-low attachment plates (Corning Incorporated, Corning, NY), where contain serum-free medium DMEM/F12 (Sigma-Aldrich) supplemented with N2 plus media supplement (Invitrogen), 20 ng/ml epidermal growth factor (Invitrogen), 20 ng/ml basic fibroblast growth factor (Invitrogen), and 4 mg/ml heparin (Sigma-Aldrich) for 14 days. The images were captured after 2 weeks of cell culture, and the number of spheres larger than 50 μm was counted.

### Cell Viability Assay

Cell viability was determined using Cell Counting Kit-8 (CCK-8) assay (Dojindo, Japan). In brief, CC cells (5000 cells per well) were seeded into 96-well plates and cultured for 24 h. The medium was refreshed and cells were exposed to varying concentrations of CDDP. Twenty-four hours after the addition of CDDP, CCK-8 solution (10 μl per well, Dojindo, Japan) was added to each well. Cells were incubated at 37°C for 3 h. The absorbance value was measured at a wavelength of 450 nm.

### Xenograft Mouse Model

The study on animal subjects was approved by the Ethics Committee of Affiliated Hospital of Inner Mongolia Medical University. *In vivo* xenograft mouse experiments were performed as previously described (14, 15). Female mice (BALB/c nude, 4–6 weeks old) were purchased from Vital River Laboratory Animal Technology (Beijing, China). CC cells transfected with HK2 overexpression vector (OriGene, Rockville, MD) or HK2 shRNA (Santa Cruz Biotechnology; Santa Cruz, CA), were subcutaneously injected into the flank of nude mice under aseptic conditions. After a 7-day administration, mice were treated with CDDP (30 mg/kg) intraperitoneally. The tumors were measured in two dimensions by using manual calipers every 3 days. The tumor volume was calculated using the following formula: volume = 0.5 × length × width^2^. After 30 days, all mice were sacrificed and tumor samples were weighted.

### Cell Transfection

Transfection was conducted using the Lipofectamine 3000 reagent (Invitrogen) according to the manufacturer’s instructions. The control vector, HK2 overexpression vector, control shRNA, or HK2 shRNA was obtained from OriGene and Santa Cruz Biotechnology, respectively. Control siRNA, HK2 siRNA, control mimic, miR-145 mimic, miR-148a mimic, miR-497 mimic, control inhibitor, miR-145 inhibitor, miR-148a inhibitor, or miR-497 inhibitor, was purchased from Invitrogen. When cells achieved 70% confluence, the above plasmid, shRNA, siRNA, or miRNA mimic (or inhibitor) was transfected into CC cells for 48 h. Finally, stable HK2-overexpressing or HK2 knockdown CC cell lines were established by selecting cells using G418 (Sigma-Aldrich) or Puromycin (Sigma-Aldrich) for 4 weeks.

### Dual-Luciferase Reporter Assay

The human *HK2* wild-type 3′-UTR luciferase reporter vector was constructed from Genechem (Shanghai, China). Mutations of the miRNA binding sites in the *HK2* 3′-UTR sequence were made using site-directed mutagenesis using the QuickChange site-directed mutagenesis kit (Stratagene, La Jolla, CA). CC cells were seeded into 24-well plates, and then co-transfected with *HK2* 3′-UTR wild-type (or mutated) luciferase reporter vector, the pRL-CMV vector (Promega, Madison, WI), along with 30 nM of miRNA mimic, control mimic, miRNA inhibitor, or control inhibitor, using Lipofectamine 3000 (Invitrogen). After 48 h, luciferase activity was examined using the Dual-Luciferase Reporter Assay System (Promega). Firefly luciferase activity was normalized to that of the Renilla luciferase activity.

### Statistical Analysis

Statistical analysis was performed using SPSS 20.0 (SPSS, Chicago). Results were presented as the mean ± standard error (SD) from three independent experiments. Statistical significance was determined using an unpaired two-tailed Student**’**s *t*-test, one-way ANOVA test, or Mann-Whitney *U* test. Pearson**’**s correlation test was used to measure the correlation coefficient between two variables. Differences were considered statistically significant if the *P*-value was < 0.05.

## Results

### Overexpression of HK2 Correlates With Poor Prognosis in CC Patients

**Figure 1:**
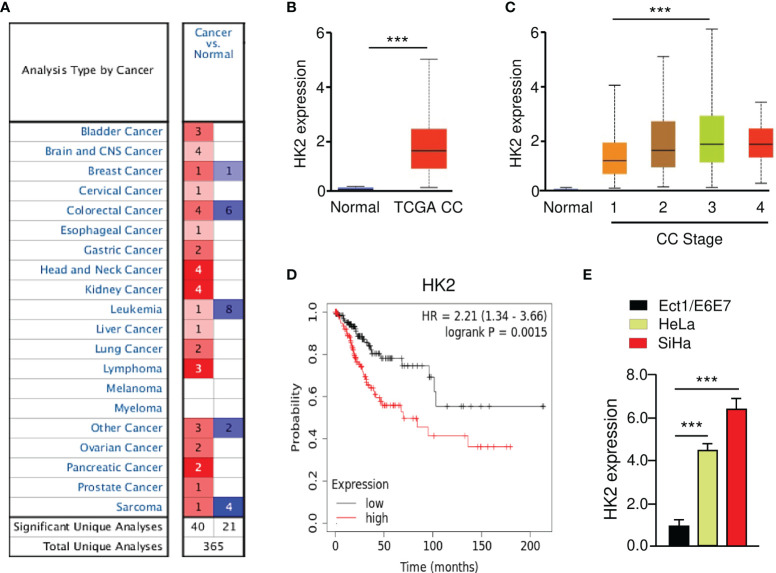
Overexpression of HK2 Correlates with Poor Prognosis in CC Patients. **(A)** The expression of HK2 in different types of cancer (Oncomine database). **(B)** The levels of HK2 in TCGA CC and normal tissues were examined using the UALCAN database. **(C)** Correlation between HK2 expression and tumor stage in CC patients (UALCAN database). **(D)** The prognostic value of HK2 in CC patients (KM plotter database). **(E)** The qRT-PCR analysis of HK2 expression in CC cells and normal ectocervical cell line Ect1/E6E7. ***P < 0.001.

The Oncomine database (https://www.oncomine.org/) was utilized to investigate the expression of HK2 between tumors and normal tissues. The results revealed that HK2 expression was elevated in most cancer tissues (including CC) compared with normal tissues (**Figure 1A**). To verify these results, we explored the expression of HK2 in TCGA CC patients using the UALCAN database (http://ualcan.path.uab.edu/index.html). The levels of HK2 were significantly increased in CC tissues than in normal tissues (**Figure 1B**). We also analyzed the correlation between HK2 expression and tumor stage in CC patients using the UALCAN database. The findings showed that those patients with advanced-stage CCs tended to exhibit higher levels of HK2 (**Figure 1C**). We evaluated the prognostic significance of HK2 in CC patients using the KM plotter database (http://kmplot.com/analysis/). Increased expression of HK2 was strongly associated with poor overall survival (**Figure 1D**). Using qRT-PCR analysis, we found an upregulation of *HK2* mRNA expression in CC cell lines (HeLa and SiHa), compared to the normal cervical epithelial cell line Ect1/E6E7 (**Figure 1E**). These results suggested that HK2 is upregulated in CC and might have a tumor-promoting function.　

### HK2 Promotes Sphere Formation and CDDP Resistance in CC Cells

**Figure 2:**
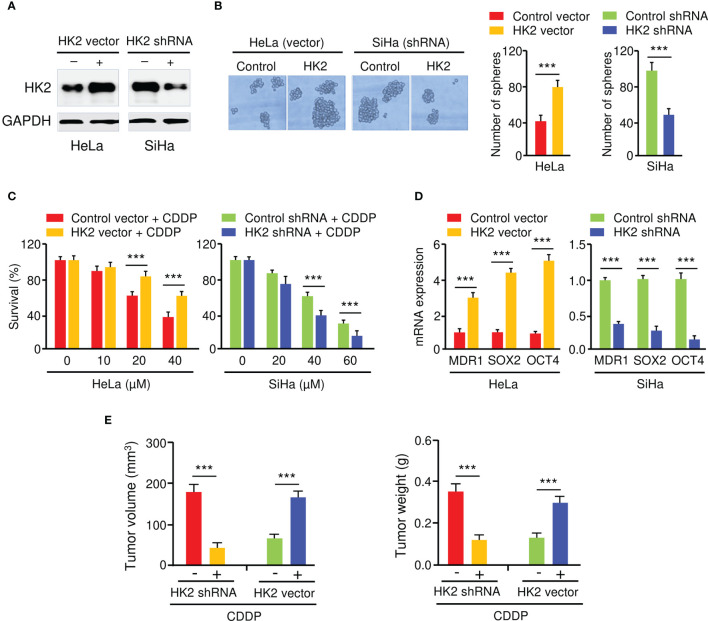
HK2 Promotes Sphere Formation and CDDP Resistance in CC Cells. **(A)** Western blot analysis of HK2 expression in CC cells transfected as indicated. **(B)** The effect of HK2 expression on the sphere formation was investigated using sphere formation assays. **(C)** Cell viability assay in response to CDDP treatment was determined using CCK-8 assay. **(D)** The expression of MDR1, SOX2, and OCT4 in CC cells transfected as indicated was detected by the qRT-PCR assay. **(E)** HK2 overexpression (or knockdown) decreased (or increased) the sensitivity of CC cells to CDDP *in vivo*. Tumor volume (left) and weight (right) were examined. ***P < 0.001.

To elucidate the biological function of HK2 in CC cells, we generated HeLa cell lines with stable HK2 overexpression and established SiHa cell lines with silencing of HK2 using HK2 shRNA. The overexpression and knockdown of HK2 in CC cells were confirmed by western blotting analysis (**Figure 2A**). The overexpression of HK2 increased the sphere formation in HeLa cells, whereas the knockdown of HK2 decreased the sphere formation in SiHa cells (**Figure 2B**). Cell viability assay showed that the overexpression (or knockdown) of HK2 significantly increased (or decreased) the resistance of CC cells to CDDP in dose-dependent assays (**Figure 2C**). The expression of *MDR1*, *SOX2*, and *OCT4* were increased after the overexpression of HK2 in HeLa cells, and an opposite effect was observed after HK2 shRNA transfection in SiHa cells (**Figure 2D**).

To analyze whether the inhibition of HK2 could sensitize CC cells to CDDP treatment *in vivo*, HK2-overexpressing HeLa cells, SiHa cells with HK2 silencing, as well as the respective control cells, were injected subcutaneously into nude mice and were treated with CDDP. Mice implanted with cells transfected with HK2 overexpression vector developed bigger tumor volumes than the control group (**Figure 2E**). Additionally, the weight of the tumors derived from the HK2-overexpressing HeLa cells was much heavier than those from the control cells (**Figure 2E**). Conversely, compared to the control mice, the mice implanted with the HK2-silenced SiHa cells had smaller tumor volumes and lighter tumor weight (**Figure 2E**). Collectively, these results suggested that HK2 overexpression facilitates the formation of spheroids and the development of CDDP resistance in CC cells.

### MiR-145, MiR-148a, and MiR-497 Are Upstream Regulators of HK2 in CC Cells

**Figure 3:**
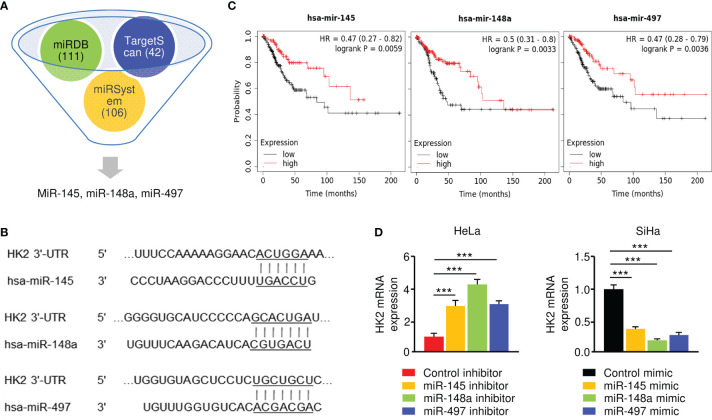
HK2 is negatively regulated by miR-145/148a/497 in CC Cells. **(A)** Prediction analysis revealed that several miRNAs might regulate HK2 expression. **(B)** The binding sites of miR-145/148a/497 in the wild-type *HK2* 3′-UTR were shown. **(C)** Survival analysis using the KM plotter database was conducted to assess the prognostic value of miR-145/148a/497 in CC patients. **(D)** The overexpression (or knockdown) of miR-145/148a/497 decreased (or increased) the mRNA expression of HK2 in CC cells. ***P < 0.001.

To investigate the upstream regulatory mechanism of HK2 in CC, we conducted *in silico* analysis to identify the potential of miRNAs to bind to *HK2* mRNA. Using TargetScan, miRDB, and miRSystem databases, a set of miRNAs (including miR-145, miR-148a, and miR-497) were found to have binding sites in the 3′-UTR of the *HK2* transcript (**Figures 3A, B**). Since the downregulation of miR-145, miR-148a, and miR-497, but not the remaining miRNAs, were correlated with poor overall survival in CC patients (**Figure 3C**), we decided to focus on these three miRNAs in our subsequent experiments.

Our qRT-PCR experiments verified that the levels of miR-145, miR-148a, and miR-497 were significantly lower in SiHa cells than in HeLa cells (data not shown). To explore the regulatory relationship between these miRNAs and HK2, we performed the qRT-PCR assay to examine the mRNA expression of *HK2* in SiHa cells transfected with miRNA mimics and in HeLa cells transfected with miRNA inhibitors. The expression of *HK2* was significantly suppressed in miR-145/148a/497 mimic-expressing SiHa cells, while the inhibition of miR-145/148a/497 upregulated *HK2* expression in HeLa cells (**Figure 3D**). These results demonstrated that HK2 is negatively regulated by miR-145/148a/497.

**Figure 4:**
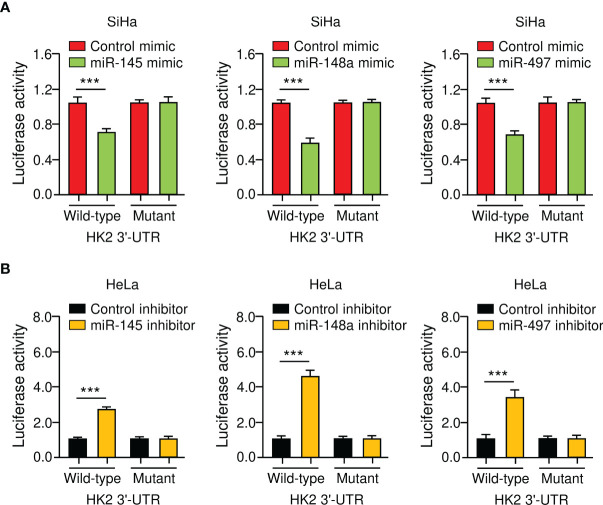
*HK2* is a Direct Target Gene of MiR-145/148a/497 in CC Cells. **(A, B)** Luciferase reporter assays of CC cells that were co-transfected with indicated miRNA mimics **(A)** or miRNA inhibitors **(B)** and luciferase vector containing the wild-type or mutated *HK2* 3′-UTR. ***P < 0.001.

To verify the direct binding between miR-145/148a/497 and *HK2* 3′-UTR, we performed luciferase reporter assays with either a wild-type or a mutated *HK2* 3′-UTR. The luciferase activity of the wild-type *HK2* 3′-UTR was suppressed by miR-145/148a/497 mimic and was enhanced by the knockdown of miR-145/148a/497 (**Figures 4A, B**). Mutations of the 3′-UTR of *HK2* abolished the effects of miR-145/148a/497 (**Figures 4A, B**). All of these results indicated that miR-145, miR-148a, and miR-497 are upstream regulators of HK2 in CC cells.

### MiR-148a Inhibits Sphere Formation and CDDP Resistance in CC Cells

**Figure 5:**
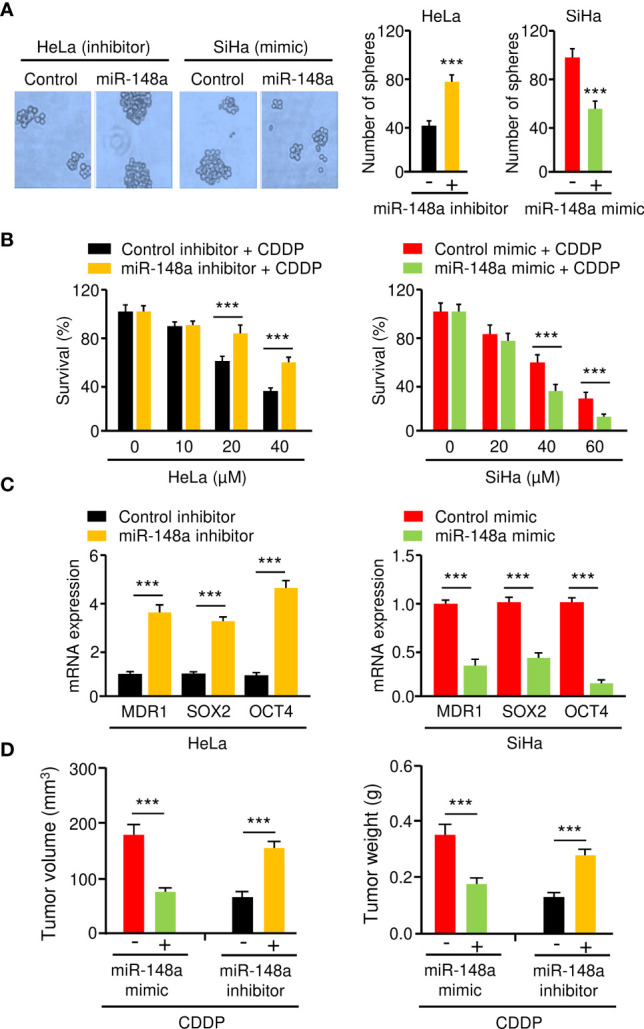
MiR-148a Inhibits Sphere Formation and CDDP resistance in CC Cells. **(A)** The effect of miR-148a expression on the sphere formation was investigated using sphere formation assays. **(B)** After transfection with miR-148a mimic or miR-148a inhibitor, CC cells were treated with CDDP and cell viability was measured using cell viability assay. **(C)** The qRT-PCR analysis of *MDR1*, *SOX2*, and *OCT4* expression in CC cells transfected as indicated. **(D)** MiR-148a overexpression (or knockdown) increased (or decreased) the sensitivity of CC cells to CDDP *in vivo*. ***P < 0.001.

MiR-145 and miR-497 are known tumor suppressors in CC (16–19). However, little is known about the role of miR-148a in CC CDDP resistance. Thus, we focused on miR-148a in this study. To analyze the effect of miR-148a overexpression or silencing on sphere formation and CDDP resistance in CC cells, we transfected miR-148a mimic into SiHa cells and introduced miR-148a inhibitor into HeLa cells. Sphere formation assays and cell viability assays showed that miR-148a overexpression significantly reduced the sphere-forming ability and attenuated CDDP resistance of SiHa cells (**Figures 5A, B**). In contrast, miR-148a inhibition significantly promoted sphere formation and increased CDDP resistance in HeLa cells (**Figures 5A, B**). The results of qRT-PCR assays confirmed the downregulation of *MDR1*, *SOX2*, and *OCT4* in miR-148a-overexpressing SiHa cells, as well as their upregulation in miR-148a-silenced HeLa cells (**Figure 5C**). To determine whether the above results were reproducible, miR-148a-overexpressing SiHa cells or miR-148a-silenced HeLa cells were injected into nude mice and were treated with CDDP. We found that the tumors formed by miR-148a-silenced HeLa cells were larger and had more weight than control tumors (**Figure 5D**). However, tumors formed by miR-148a-overexpressing SiHa cells were smaller in both size and weight than the tumors formed by control cells (**Figure 5D**). Thus, we demonstrated that miR-148a serves as a tumor suppressor by antagonizing sphere formation and increasing the CDDP sensitivity of CC cells.

### The MiR-148a/HK2 Axis Mediates Sphere Formation and CDDP Resistance in CC Cells

**Figure 6:**
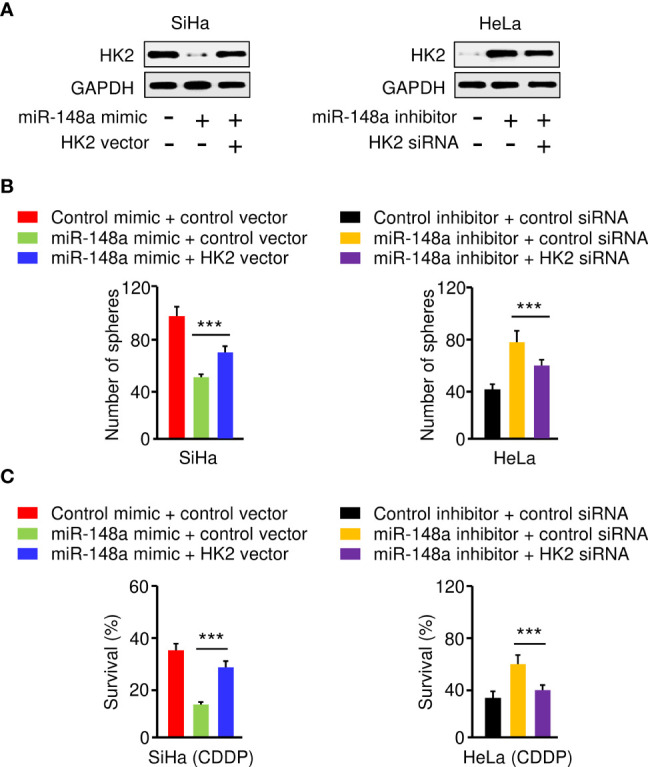
The MiR-148a/HK2 Axis Mediates Sphere Formation and CDDP Resistance in CC Cells. **(A)** Western blot analysis of HK2 expression in CC cells transfected as indicated. **(B)** Sphere formation of CC cells transfected as indicated. **(C)** Cell viability of CC cells transfected as indicated in the presence of CDDP was detected by the cell viability assay. ***P < 0.001.

To investigate whether miR-148a suppresses sphere-forming ability and CDDP resistance of CC cells by targeting HK2, we transfected SiHa cells with miR-148a mimic (or control mimic), along with HK2 overexpression vector (or control vector), and also transfected HeLa cells with miR-148a inhibitor (or control inhibitor), together with HK2 siRNA (or control siRNA). The protein expression of HK2 was examined by western blotting analysis. The expression of HK2 was decreased with the transfection with miR-148a mimic, and this reduction was reversed by the introduction of the HK2 expression vector (**Figure 6A**). In addition, the protein expression of HK2 was induced by miR-148a inhibition, and this increase was reversed by transfection with HK2 siRNA (**Figure 6A**). The results of sphere formation and cell viability assays showed that sphere formation and CDDP resistance were suppressed by transfection of miR-148a mimic, whereas this repression could be eliminated by forced expression of HK2 (**Figures 6B, C**). We also verified that miR-148a inhibition induced sphere formation and CDDP resistance, while knockdown of HK2 attenuated these malignant properties of CC cells (**Figures 6B, C**). Consequently, these results demonstrated that the miR-148a/HK2 axis functionally regulates sphere formation and CDDP resistance in CC cells.

### Correlation Between MiR-145/148a/497 and HK2 Expression in CC Tissues

**Figure 7:**
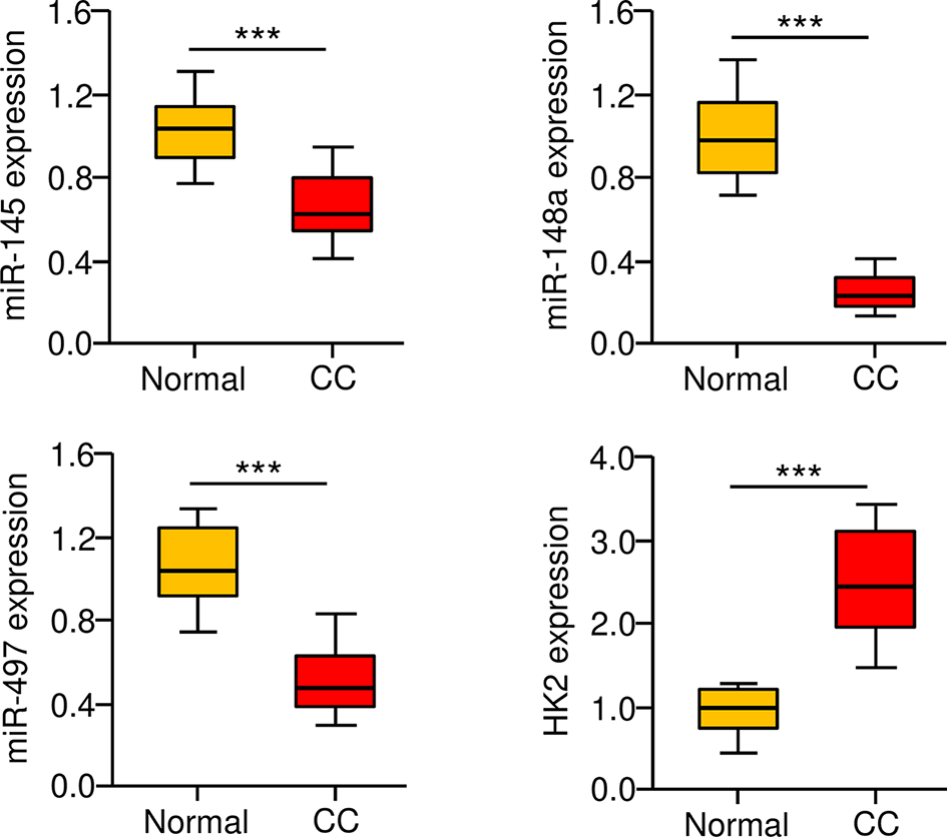
Correlation between MiR-145/148a/497 and HK2 Expression in CC Tissues. The expression of miR-145, miR-148a, and miR-497 in CC and adjacent normal tissues was measured using the qRT-PCR assays. ***P < 0.001.

**To** further define the role of miR-145, miR-148a, miR-497, and HK2 in human CC, we measured their expression in 30 adjacent normal tissues and 30 CC tissues using the qRT-PCR assay. We found that CC tissues exhibited lower expression of miR-145, miR-148a, and miR-497 (**Figure 7A**). Our results also showed increased expression of HK2 in human CC tissues (**Figure 7**). Overall, these results support the notion that HK2 is crucial for the maintenance of the sphere-forming property and CDDP resistance in CC cells, and upregulation of HK2 observed in CC tissues might be caused by the repression of multiple tumor suppressor miRNAs (including miR-145, miR-148a, and miR-497).

## Discussion

Even though CDDP has been certified as a front-line drug for CC treatment, primary and acquired CDDP resistance leads to the failure of CDDP-based therapy and accounts for the recurrence of CC (2). Growing evidence has indicated that cancer stem cells could contribute to chemoresistance through various mechanisms, such as induction of metabolic enzyme aldehyde dehydrogenase (20), overexpression of ABC transporters (21), activation of Notch, Hedgehog, and Wnt pathways (22). In this work, we have demonstrated that HK2, a critical enzyme in glucose metabolism, is commonly overexpressed in human CC, and knocking down HK2 sensitizes CC cells to CDDP. Besides, overexpression of HK2 could be partially due to the downregulation of miRNAs (especially miR-148a). To our knowledge, this study is the first to elucidate the oncogenic role of HK2 in promoting CDDP resistance in CC, and to reveal the biological relevance of the miR-14a/HK2 axis in the sphere formation ability and CDDP sensitivity of CC cells.

Aberrant expression of HK2 in CC has been previously reported (8, 9). The results from our meta-analysis and qRT-PCR assays have confirmed the elevated expression of HK2 in CC tissues and cell lines, and the upregulation of HK2 is closely associated with worse survival rates of CC patients. Although the silencing of HK2 was known to inhibit the growth and migration of CC cells (8, 9), whether HK2 could influence the sphere-forming properties and the development of CDDP resistance of CC cells remains unknown. Our gain-of-function and loss-of-function assays have uncovered the promoting role of HK2 in maintaining cancer stem cell-like ability and the acquisition of CDDP resistance. Importantly, knockdown of HK2 combined with CDDP treatment reduces CC proliferation *in vivo*. These results together suggested that targeting HK2 may abrogate cancer stem cell populations, as demonstrated by reduced sphere formation capacity, and regain the sensitivity of CC patients to CDDP.

In addition to regulating glucose metabolism, HK2 promotes cancer stem cell self-renewal in CC cells, elucidating that HK2 is an upstream activator of cancer stem cell expansion (23). In ovarian cancer, overexpression of HK2 enhances stemness properties by upregulating the expression of cancer stemness-related genes, such as OCT4 (24). Moreover, HK2 is required for the maintenance of esophageal cancer stem cell phenotypes (25). The chemoresistance to 5-FU and CDDP and the tumorigenesis of esophageal cancer stem cells are reduced after HK2 knockdown (25). Overexpression of HK2 effectively promoted epithelial-mesenchymal transition (EMT) phenotypes and enhanced aerobic glycolysis in EC cells (26). Consistent with these previous studies, the current demonstrated that silencing HK2 decreases the expression of cancer stem cell markers OCT4 and SOX2, suggesting that therapeutic approaches targeting HK2 might be an effective strategy for suppressing the expansion and maintenance of the cancer stem cell population in CC cells.

Targeting HK2 using HK2 inhibitors has been a hopeful strategy for cancer treatment. To date, different types of HK2 inhibitors have been developed (27). For instance, the selective HK2 inhibitor Benitrobenrazide binds directly to HK2, blocks glycolysis, and induces apoptosis in HK2-overexpressing cancer cells (28). Glycolysis is activated in radioresistant CC cells, and inhibiting glycolysis with HK2 inhibitor 2-DG improves the sensitivity of radioresistant CC cells to irradiation (29). These results support the possibility that inhibition of glycolysis *via* specific HK2 inhibitors, in combination with other therapies, would be an effective strategy for the treatment of CC.

Having established the role of HK2 in sphere formation and CDDP resistance of CC cells, we sought mechanistic evidence for activation of HK2. According to our *in silico* analysis, qRT-PCR assays, and luciferase reporter assays, three miRNAs (miR-145, miR-148a, and miR-497) were determined as upstream regulators of HK2 in CC cells. MiR-145 and miR-497 suppress the aggressive phenotypes of CC cells (16–19). MiR-145 negatively modulates proliferation, EMT, migration, and invasion of CC cells (16). Interestingly, miR-145 directly binds to *HK2* mRNA and reduces its expression in renal cell carcinoma (30). In our work, we have found that miR-145, miR-148a, and miR-497 inhibit endogenous HK2 expression by binding to its 3′-UTR. Consequently, silencing of these miRNAs in CC tissues stimulates the expression of HK2, facilitating the development of cancer stem cell-like properties and CDDP resistance. Collectively, we propose a complex regulatory relationship between these miRNAs and HK2 in CC. Of note, HK2 levels are regulated at the transcriptional, post-translational, and translational levels (31). Different genetic and epigenetic mechanisms may be involved in the regulation of HK2 in CC, the exact mechanisms for the upregulation of HK2 are deserving of future studies.

Aberrant expression of miR-148a is a common event in human tumors (32). For instance, the downregulation of miR-148a has been detected in various cancers, including gastric, colon, pancreatic, liver, esophageal, breast, and lung cancer (32). It is known that miR-148a could suppress the proliferation, migration, invasion, and metastasis of cancer cells by targeting a large number of downstream genes (32). Of note, in esophageal cancer cells, re-expression of miR-148a significantly improves the sensitivity of cells to CDDP and 5-FU (33). In cervical cancer, a previous study using miRNA array and qRT-PCR assay has suggested that miR-148a levels were significantly lower in CC tissues compared to normal tissues (34). Overexpression of miR-148a could markedly suppress the proliferation of CC cells (35). Moreover, lncRNA SNHG4 promotes the proliferation of CC cells through regulating c-Met *via* targeting miR-148a (36). LncRNA SNHG12 sponges miR-148a to increase CDK1 expression, thereby attenuating the radiation-induced apoptosis in CC cells (37). In line with this research, we have shown that miR-148a attenuates CDDP resistance in CC cells, at least in part, by targeting HK2. Therefore, our results elucidated a new mechanism for miR-148a in chemoresistance. Cancer cells frequently overexpress some proteins implicated in glucose metabolism, such as GLUT1, the main glucose transporter (38). Since a recent study has reported that GLUT1 is a direct target of miR-148a in human intrahepatic cholangiocarcinoma (39), miR-148a may also regulate glucose metabolism by inhibiting GLUT1 in CC. Future studies to identify the interactions between miR-148a and its targets would strengthen our understanding of the function of miR-148a, and yield key insights into the metabolic landscape of CC.

## Conclusion

**Figure 8:**
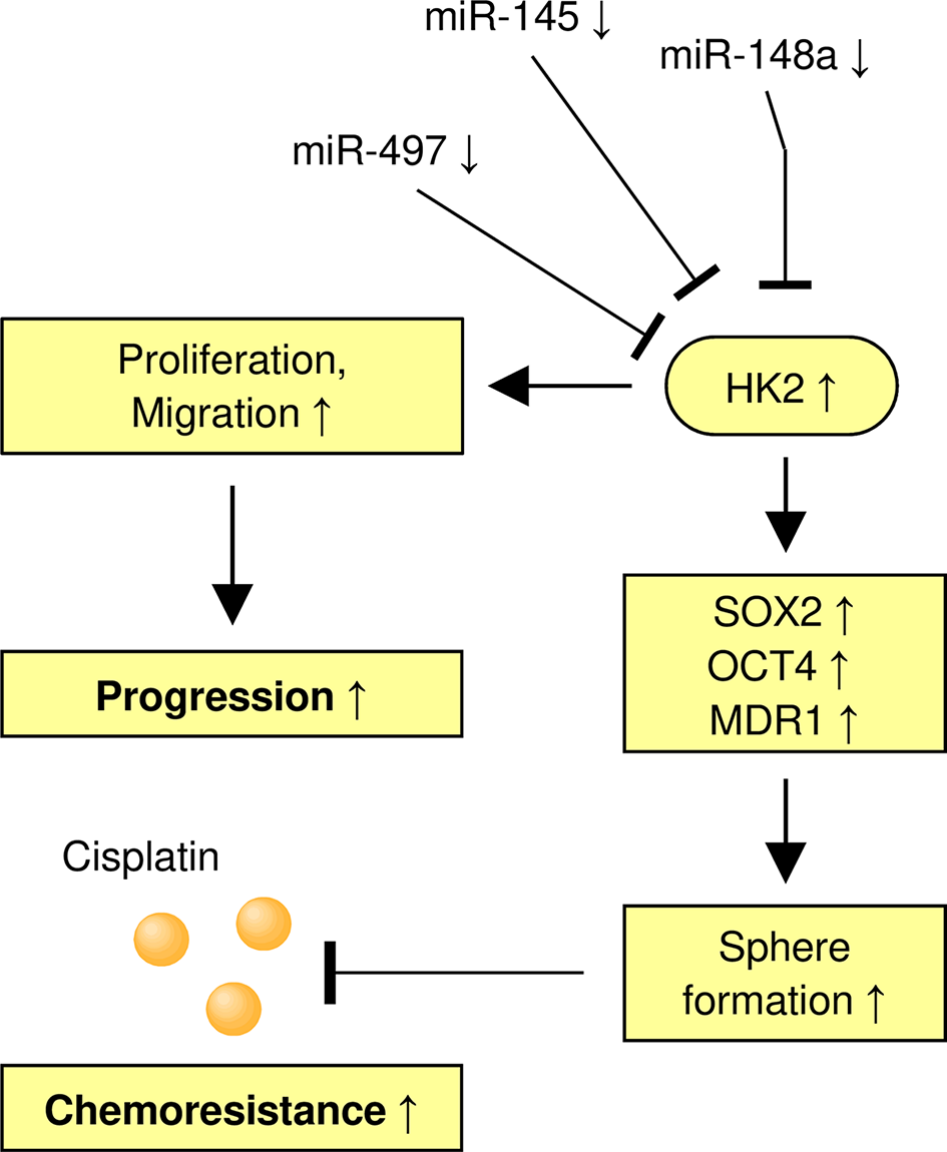
A Model Shows the Role of the MiR-148a/HK2 Axis in Regulating Sphere Formation and CDDP Resistance of CC Cells.

In summary, our findings demonstrate the involvement of HK2 in the maintenance of the sphere-forming property and CDDP resistance in CC cells. In addition, we have identified miR-148a as an upstream suppressor of HK2 to inhibit the ability of sphere formation and increase the sensitivity of CDDP in CC cells (**Figure 8**). We have uncovered a previously unknown miR-148a/HK2 axis that modulates cancer stem cell-like features and CDDP resistance in CC.

## Data Availability Statement

The original contributions presented in the study are included in the article/supplementary material. Further inquiries can be directed to the corresponding authors.

## Ethics Statement

The studies involving human participants were reviewed and approved by Research Ethics Committee of Affiliated Hospital of Inner Mongolia Medical University. The patients/participants provided their written informed consent to participate in this study. The animal study was reviewed and approved by Ethics Committee of Affiliated Hospital of Inner Mongolia Medical University.

## Author Contributions

HZ and TY designed the experiments. HY and HH conducted the experiments. TY, YCH, YH, and JG analyzed the data. All authors contributed to the article and approved the submitted version.

## Funding

This study was supported by a grant from the Natural Science Foundation of China (81860534), Inner Mongolia autonomous region science and technology planning project (2019GG039, 2019GG086, 2021GG0167), Zhi Yuan Talent Projects of Inner Mongolia Medical University (ZY0202011), Natural Science Foundation of Inner Mongolia Autonomous Region of China (2021MS08152), Xisike-Shiyao Clinical Oncology Research Foundation(Y-SY201901-0008), and Xisike-Qilu Clinical Oncology Research Foundation (Y-QL2019-0137).

## Conflict of Interest

The authors declare that the research was conducted in the absence of any commercial or financial relationships that could be construed as a potential conflict of interest.

## Publisher’s Note

All claims expressed in this article are solely those of the authors and do not necessarily represent those of their affiliated organizations, or those of the publisher, the editors and the reviewers. Any product that may be evaluated in this article, or claim that may be made by its manufacturer, is not guaranteed or endorsed by the publisher.
